# Natural β-carotene prevents acute lung injury induced by cyclophosphamide in mice

**DOI:** 10.1371/journal.pone.0283779

**Published:** 2023-04-05

**Authors:** Farouk K. El-Baz, Sami I. Ali, Rania Elgohary, Abeer Salama

**Affiliations:** 1 Plant Biochemistry Department, National Research Centre (NRC), Cairo, Egypt; 2 Narcotics, Ergogenics and Poisons Department, National Research Centre (NRC), Cairo, Egypt; 3 Pharmacology Department, National Research Centre (NRC), Cairo, Egypt; University of Lille, FRANCE

## Abstract

IL-17 is associated with varied inflammatory and immune-related diseases. However, the biological function of IL-17 and its expression in acute lung damage are not entirely known. Thanks to the powerful antioxidant properties of β-carotene, we presumed that it would show a potent protecting effect against cyclophosphamide (CP) -induced acute lung injury (ALI) in mice. We studied the mechanisms underlying the effect of β-carotene supplementation against CP-induced ALI in mice. We isolated the β-carotene from *Scenedesmus obliquus* microalgae n-hexane extract and identified it by HPLC and ^1^H-NMR analysis. Within the experiments, 40 mice were assigned into five groups randomly: Group 1 (Control): Mice received saline. Group 2 (β-carotene control): Mice were administered β-carotene (40 mg/kg; orally) once daily for 10 sequent days without CP injection. Group 3 (CP): One i.p injection of 200 (mg/kg) of CP was given to mice. Group 4 and 5 (CP + β-carotene): Mice were administered β-carotene (20 and 40 mg/kg; orally) once a day for ten days following the CP injection. Lung samples were collected for lab analysis, after scarifying the animals at the experiment end. Administration of β-carotene orally reduced CP-induced ALI and inflammation. β-carotene significantly decreased wet-to-dry weight ratios (W/D), down-regulated IL-17, NF-κB, and IKBKB, decreased the contents of TNF-α, COX-2, and PKC, and increased the contents of SIRT1 and PPARγ in the lung tissues. β-carotene ameliorated the histopathological changes induced by CP and reduced the scoring number of inflammatory cell infiltration and emphysema when compared to CP. Consequently, we conclude natural β-carotene is a promising anti-inflammatory mediator for different inflammatory-related complications.

## Introduction

Carotenoids are lipophilic ingredients that are characterized by their chemical structure composed of a 40-carbon backbone with a large, conjugated double-bond structure. Thanks to their potent antioxidant capacity to quench singlet oxygen, they can inhibit the progress of different human ailments, for instance cancer, age-related macular degeneration, inflammation, and immune-related and cardiovascular diseases [1]. Microalgae are a prominent renewable producer of beta-carotene and other carotenoids [2–4]. Green algae of the genus Scenedesmus including *Scenedesmus obliquus* can synthesize adequate amounts of β-carotene [5, 6]. Beta-carotene, one of the most widely consumed carotenoids, is characterized by its high-fat solubility, a precursor for vitamin A biosynthesis, effective antioxidant ability including free radical hunting, singlet oxygen quencher, and an ability to inhibit lipid peroxidation [7]. Former epidemiological studies recommended the dietary intake of daily beta-carotene to protect against diabetes, cardiovascular diseases, rheumatoid arthritis, atherosclerosis, inflammatory bowel disease, and cancer [1, 8]. Former *in vivo* results [9] have shown that administration of β-carotene significantly reduced the degree and the harshness of the hemorrhages and inflammation features (arterialization of the small venules, obvious inflammation of the small arteries and arterioles, abundant deposition of collagen in the septa, and severe focal pneumonia) in monocrotaline-treated rats. In another study [10], β-carotene and 9-cis-carotene-rich diet, supplied as Dunaliella microalgae powder, prevented atherosclerosis, and lessen inflammation in the livers of mice by reducing the expression of different genes VCAM-1, IL-1a, E-selectin, and TLR2 against the control. Our previous study [4] reported the potent ability of β-carotene and zeaxanthin-enriched fractions of *Dunaliella salina* to reduce inflammations in carrageenan-induced inflammation in rats through the downregulation of proinflammatory cytokines IL-6, TNF-α, COX-2, and PGE2 and the neurotransmitter serotonin.

Cyclophosphamide (CP) alkylating agent is used as an immunosuppressive in organ transplantation and for neoplastic diseases, but its use is accompanied by several organ toxicities [11]. CP can induce lung toxicity which may enhance lung fibrosis [12] via oxidative, inflammatory, and fibrotic reactions. Acrolein, the toxic metabolite of CP, showed a high ability to alkylate the DNA and other cellular structures [13]. Oxidative stress is implicated in CP-induced pulmonary damage. Overproduction of reactive oxygen species (ROS) stimulates severe infiltration of inflammatory cells including macrophages, neutrophils, and monocytes resulting in pulmonary fibrosis [14]. Additionally, the generation of ROS stimulates nuclear factor kappa-β (NF-kβ) along with other signaling factors, which alters the inflammatory cascade [15, 16] through the gene expression of cyclooxygenase-2 (COX-2), inducible nitric oxide synthase (iNOS), and pro-inflammatory cytokines [17]. T-helper-17 cells are associated with the pathogenesis of autoimmune and inflammatory disorders including chronic obstructive pulmonary, cystic fibrosis, and disease asthma [18]. T-helper cells are associated with pro-inflammatory IL-17 and IL-22 release [19]. In addition, IL-17 plays a vital role in immunity enhancement against various bacterial infections through the activation and recruitment of neutrophils [20]. In ALI, circulating and alveolar IL-17 levels are elevated in humans with acute respiratory distress syndrome (ARDS) [21]. In addition, macrophages and neutrophils are important mediators in inducing pro-inflammatory factors such as TNF-α, IL-6, and IL-1b, and mediating ALI/ARDS [22].

The earlier findings of the *in vitro* and *in vivo* experiments that confirm the potent health benefits of β-carotene encouraged us to evaluate the effect and mechanisms involved after pretreatment with natural β- carotene in ALI induced by CP through multiple proinflammatory markers in lung tissue. Knowing the importance of NF-kβ /IL-17 signaling in ALI may be important as a future therapeutic target for this disease. As well the isolation and NMR structural elucidation of β-carotene from the n-hexane extract of *S*. *obliquus* microalgae will be investigated.

## Materials and methods

### Cultivation of *S*. *obliquus*

*S*. *obliquus* was isolated from the freshwater community of the River Nile in October 2011 and grown on BG11 media [23]. Cultivation was conducted in plastic bottles with a capacity of 17 L having 15 L of microalgal culture with continuous aeration. The culture temperature was 22 ± 3°C. Fluorescent light was used to supply a constant light intensity ≈of 2,500 lux for the culture. After 10 days of algal growth, the culture was transferred to a fully automated and computer-controlled vertical photobioreactor with a capacity of 4000 L. Carbon dioxide was injected into the culture as a carbon source. The culture was left to grow until the biomass reached 2–2.5 g/L. Algal biomass was harvested by centrifugation at 2000 rpm for 15 min using a basket centrifuge. Samples were washed twice with water, dried in an oven at 50 °C, ground into a homogenous powder, and stored in a deep freezer until used.

### Preparation of microalgal extract

The fine powder of *S*. *obliquus* (200 g) was extracted with n-hexane (2 L x 3) for 24 h, centrifuged (Sigma 3-18ks Centrifuge, Germany) at 5000 rpm for 20 min at 25°C to separate cell debris from the supernatant. The supernatants evaporated under a vacuum (Heidolph Unimax 2010, Germany) at 40°C to obtain n-hexane extract (8.77g). All the extraction steps were performed in dim light [3].

### Isolation of β-carotene

An aliquot of the n-hexane extract (4 g) was dissolved in 20 mL n-hexane and kept in the refrigerator overnight to precipitate fatty acids. To purify β-carotene, the n-hexane soluble portion was subjected to vacuum liquid chromatography (VLC) over 150 g of 40–63 mesh silica gel (Sigma-Aldrich) and eluted with n-hexane (1.5 L) yielding the first fraction (F1, the yellow band contains β-carotene, 1.3 g), then with acetone (1 L) yielding the second fraction (F2, 2.5 g). HPLC analysis revealed that F1 is enriched with β-carotene when compared with the β-carotene standard (Sigma-Aldrich), and F2 is a mixture of other carotenoids. F1 (orange oily structure) was further purified by using preparative TLC (Merck, F254 glass plates 20X20cm, 50μm) using 1% acetone in hexane as the mobile phase to obtain an amorphous orange powder (0.2 g) of β-carotene. The chemical structure of β-carotene was confirmed by ^1^H-NMR analysis.

### HPLC analysis

The HPLC analysis of n-hexane extract and its fractions (F1 and F2) was performed using an Agilent 1260 infinity series HPLC-DAD system (Agilent Technologies, Waldbronn, Germany) equipped with a binary gradient Agilent 1260 prep pump (G1361A) and an autosampler Agilent 1260 prep ALS (G2260A). Agilent diode array detector 1260 DAD VL (G1315D) was employed for the detection of carotenoids. The separation was performed using an Agilent normal phase (NP) silica column (ZORBAX RX-Sil, 5μm, 4.6 X 150 mm). The following solvents (A) n-hexane and (B) acetone were used at a flow rate of 1 mL/min using a gradient between solvents A and B following the method of Prum et al. [24] with some modifications as follows: B was run at 0 to 30% for 5 min, 30 to 50% for 15 min, 50 to 100% for 3 min, and maintaining 100% of B until the end of the separation at 30 min. The peaks were integrated at 450 nm. β-carotene (Sigma-Aldrich) was used as a standard to identify the isolated β-carotene.

### Identification of β-carotene from *S*. *obliquus* by Nuclear Magnetic Resonance (NMR)

The chemical structure of β-carotene isolated from *S*. *obliquus* was confirmed by using ^1^H-NMR analysis on an NMR spectrometer (500 MHz, JEOL, USA) using CDCl_3_ (Merck, Germany) as the solvent. The chemical shifts are reported in ppm (parts per million; δ) and coupling constants (J) are expressed in Hz. TMS was used as an internal standard. For improving the signal-to-noise ratio. The data were analyzed using the software program MestReNova v8.0.2 (2012 Mestrelab Research S. L.).

### Animals

Adult male Swiss mice weighing 30–40 g were purchased from the animal house colony of the National Research Centre (Dokki, Giza, Egypt) and were kept at room temperature 25 °C ± 2 and with a 12-h on/off the light program. During the whole experiment, standard food and water were provided to animals under conventional laboratory conditions. Mice were allowed to adapt to these conditions for 2 weeks before beginning the experimental protocol. All experimental procedures were conducted according to the ethical principles and guidelines of the use, care, and handling of experimental animals adopted by the Medical Research Ethics Committee (MREC) at the National Research Centre, Egypt (Reg. No. 19/116), which is by the Principles of Laboratory Animal Care (NIH No. 85:23 revised 1985) by the guidelines provided by the CPCSEA and World Medical Association Declaration of Helsinki on Ethical Principles for studies involving experimental animals. The animals were sacrificed under sodium pentobarbital anesthesia, and all efforts were made to minimize suffering. All experimental procedures were conducted in compliance with the Animal Research: Reporting of In Vivo Experiments (ARRIVE) guidelines.

### Chemicals and kits

Cyclophosphamide (CP) was purchased from (Santa Cruz Biotechnology, USA). Sirtuin 1 (SIRT1), was determined using ELISA kits procured from (SunRed Biotech Co., Ltd, China). Tumor necrosis factor alpha (TNF-α), Interleukin-17 (IL-17), and Peroxisome proliferator-activated receptor gamma. (PPARγ), Cyclooxygenase-2 (COX-2), Nuclear factor kappa B (NF-κB), Inhibitor of nuclear factor kappa b kinase subunit beta (IKBKB), and Protein kinase C (PKC) were determined using ELISA kits procured from (Sunlong Biotech Co., Ltd, China).

### Experimental design

The 40 male Swiss mice were randomly allocated into five groups (n = 8) as follows: (1) Normal control group: Mice were injected with a single i.p injection of normal saline and received normal saline orally for 10 consecutive days, (2) β-carotene control group: Mice were administered only β-carotene (40 mg/kg; orally) once daily for 10 consecutive days, (3) Cyclophosphamide (CP) group: Mice were injected with a single i.p injection of CP (200 mg/kg) [25], and (4 and 5) β-carotene groups: Mice were administered β-carotene (20 and 40 mg/kg; orally) [26] once daily for 10 consecutive days after CP injection.

### Wet-to-dry weight (W/D) ratio assay

At the end of the experimental period, the animals were sacrificed by decapitation, the lung from mice was immediately dissected, blood and other contaminants were removed, the right upper lung tissue was weighed, dried in a drying oven at 60°C for 72 hours, and weighed again according to the method of Oliveira [27]; the W/D ratio is expressed as the ratio of wet lung weight (mg) to dry lung weight (mg).

### Biochemical analysis

The lung tissue was immediately washed with ice-cooled physiological saline and homogenized in phosphate-buffered saline (PBS, pH 7.4) at 20% (w/v) for the biochemical measurements of SIRT1, TNF-α, IL-17, NF-κB, IKBKB, PPARγ, COX-2, and PKC using ELISA kits.

### Histological examination

The dissected lungs of diverse groups were fixed in 10% formalin. Fixation for one or two days was followed by dehydration in ascending grades of alcohol (70%, 90%, and three changes in absolute alcohol), clearance with xylene, impregnation in three successive changes in soft paraffin at 50°C, and finally embedded in paraffin wax to obtain solid blocks containing the tissue. Serial transverse sections of 7 μm thick were cut. Paraffin sections were mounted on glass slides covered by albumin glycerin and then stained with Hematoxylin and Eosin. Hematoxylin and Eosin sections were evaluated qualitatively under light microscopy [28]. The extent of inflammatory cell infiltration and emphysema was quantified based on previous studies [29]. Whole-lung sections were scored by observers who were blinded for groups. The degree of inflammatory cell infiltration and bronchiolar epithelium hyperplasia were assessed in alveolar septa and lumens. The scores were derived using light microscopy and scored based on the intensity of alterations: 0, absent; 1, mild; 2, moderate; 3 severe. Scores of rats from the same group were pooled and calculated as mean ± standard error of the mean (SEM).

### Statistical analysis

All the values are presented as means ± SE. Data of this study were evaluated by one-way analysis of variance followed by Tukey’s multiple comparisons tests. Graph pad Prism software, version 5 (Inc., San Diego, USA) was used to conduct these statistical tests. The difference was considered significant when *p<0*.*05*.

## Results

### Extraction and identification of β-carotene from *S*. *obliquus* microalgae

**Fig 1:**
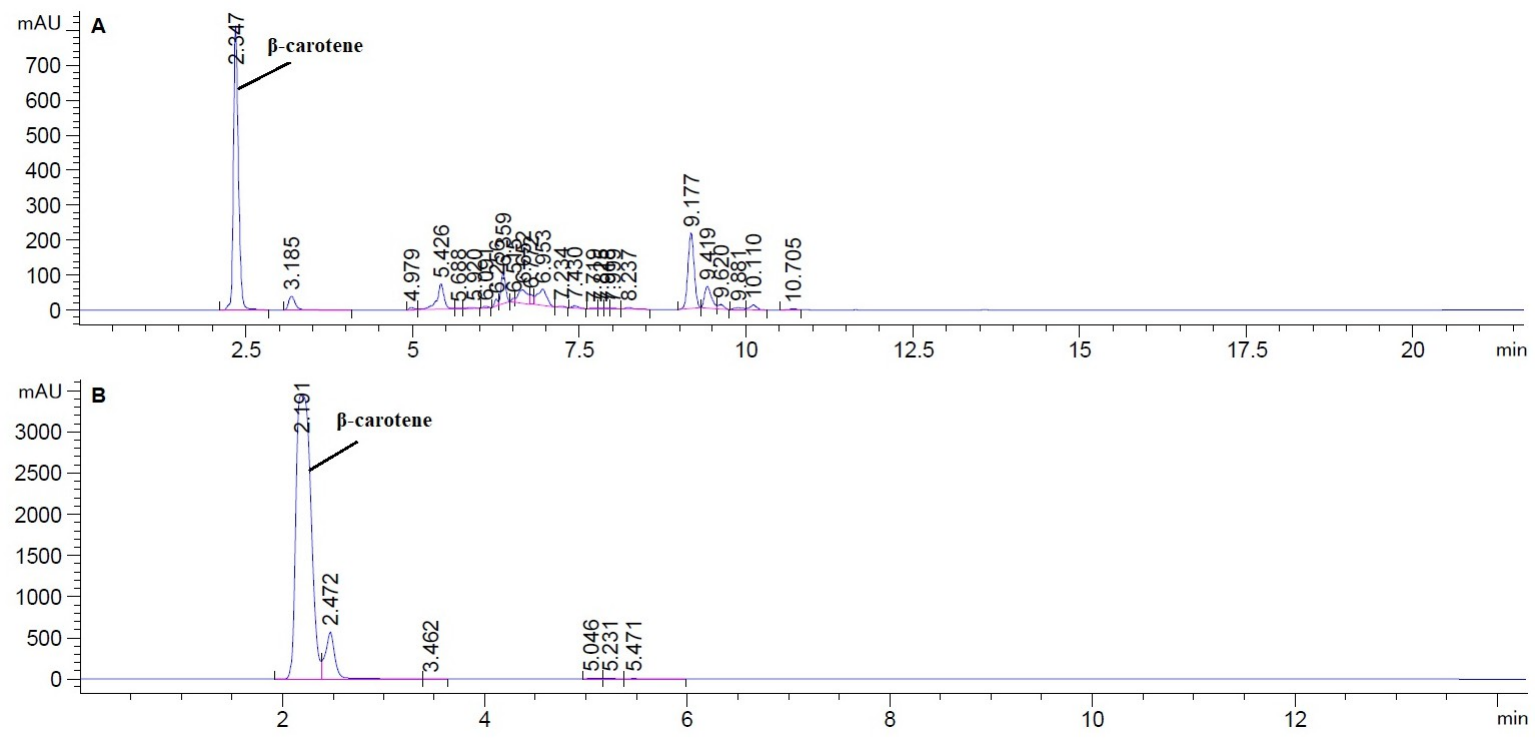
HPLC chromatogram of n-hexane of *S*. *obliquus* (A), and the yellow band containing β-carotene F1 (B).

**Fig 2:**
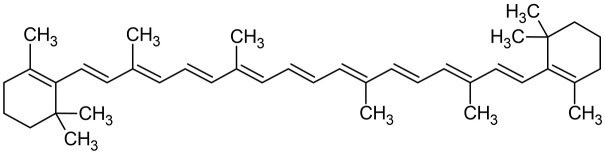
Chemical structure of β-carotene.

**Fig 3:**
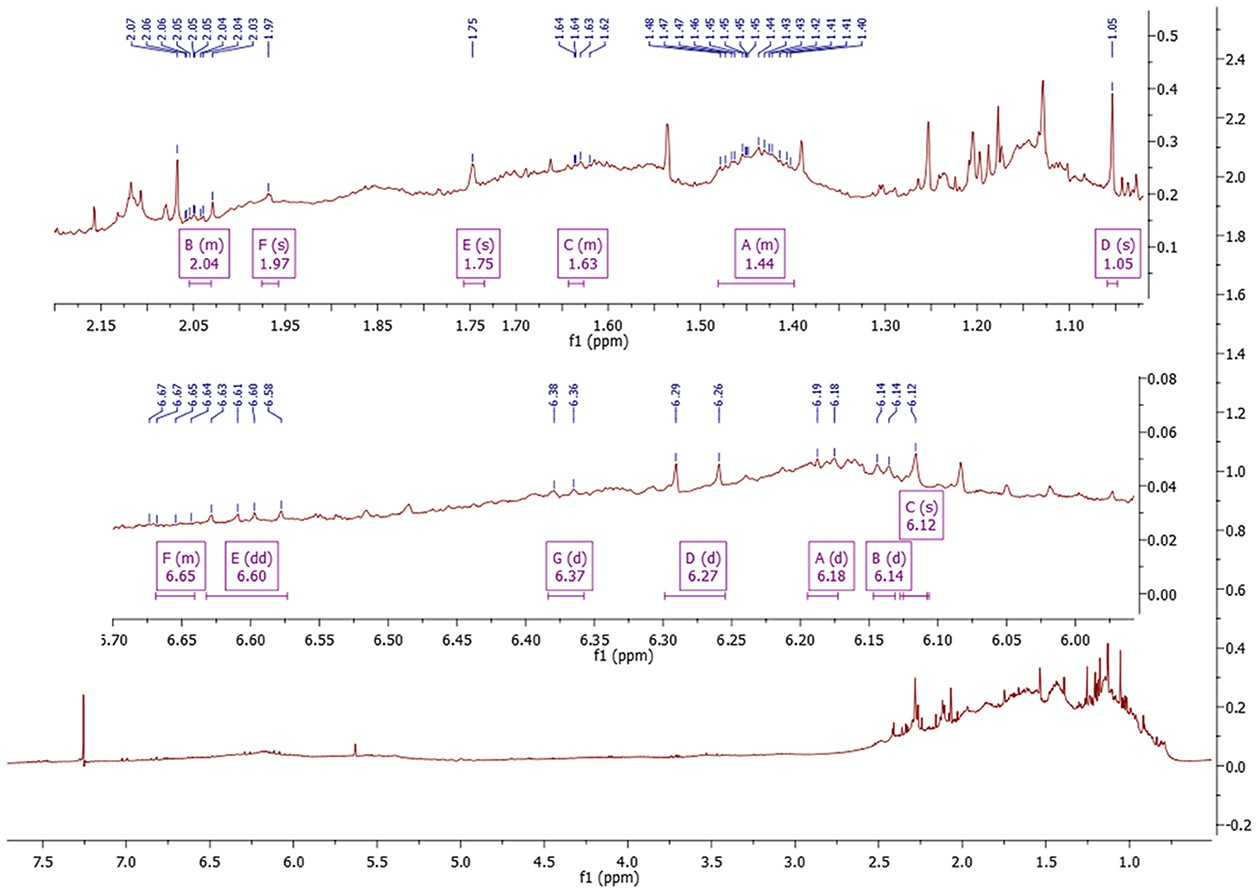
The ^1^H NMR spectrum of β-carotene isolated from *S*. *obliquus* microalgae. (500 MHz, CDCl_3_).

The hexane was used for carotenoid extraction from *S*. *obliquus*, yielding 8.77g of the crude extract with an extract yield of 4.385%. The HPLC analysis of n-hexane extract showed an abundant peak at 2.347 min representing 39.82% of the total peak area at 450 nm (Fig 1A). The yellow band containing β-carotene (F1) was obtained by VLC eluted with n-hexane, F1 showed an oily structure in a mixture with other carotenoids and unsaturated fatty acids (Fig 1B). The Prep TLC was used for the entire purification of β-carotene giving an orange powder of β-carotene. The chemical structure of β-carotene (Fig 2) was identified based on the HPLC analysis and confirmed by ^1^H-NMR (Table 1 and Fig 3).

**Table 1:** The ^1^H- NMR spectroscopic data of β-carotene isolated from *S*. *obliquus* microalgae.

Position	δ_H_ (500 MHz, J values in Hz)
1, 1′	-
2, 2′	1.44 (m, 4H)
3, 3′	1.63 (m, 4H)
4, 4′	2.04 (m, 4H)
5, 5′	-
6, 6′	-
7, 7′	6.18 (d, J = 6.2 Hz, 2H)
8, 8′	6.14 (d, J = 4.3 Hz, 2H)
9, 9′	-
10, 10′	6.27 (d, J = 15.7 Hz, 2H)
11, 11′	6.60 (dd, J = 15.7, 9.6 Hz, 2H)
12, 12′	6.37 (d, J = 7.2 Hz, 2H)
13, 13′	-
14, 14′	6.12 (s, 2H)
15, 15′	6.65 (m, 2H)
16, 16′	1.05 (s, 6H, Me)
17, 17′	1.05 (s, 6H, Me)
18, 18′	1.75 (s, 6H, Me)
19, 19′	1.97 (s, 6H, Me)
20, 20′	1.97 (s, 6H, Me)

### Wet-to-dry weight (W/D) ratio results

**Fig 4:**
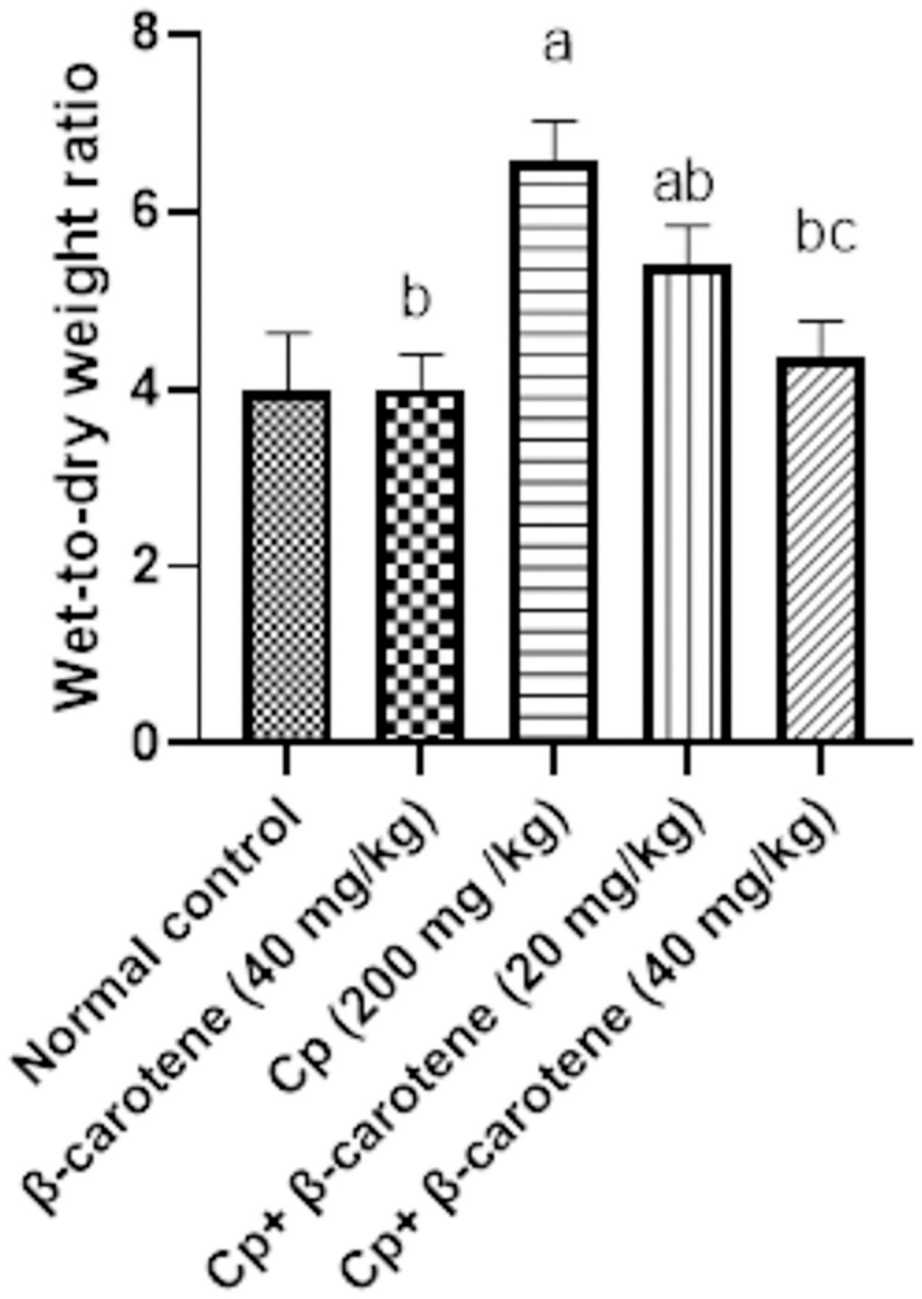
Effect of treatment with β-carotene on wet-to-dry weight (W/D) ratio. ^a^Significantly different from the control group. ^b^Significantly different from the CP group. ^c^Significantly different from the CP + β-carotene 20 mg/kg group at P<0.05.

At 10 days, the W/D ratios were 3.98 ± 0.061, 4.01 ± 0.039, 6.58± 0.043, and 5.42 ± 0.042, and 4.38 ± 0.038 in the control, β-carotene normal, CP, β-carotene 20 and β-carotene 40 groups, respectively. The high W/D ratio in the CP group indicates the induction of acute lung injury (ALI) in mice by CP injection. Administration of β-carotene 20 and 40 mg/kg lowered the W/D ratio as compared to the CP group (Fig 4).

### β-carotene supplementation decreased NF-κB and IKBKB lung contents

**Fig 5:**
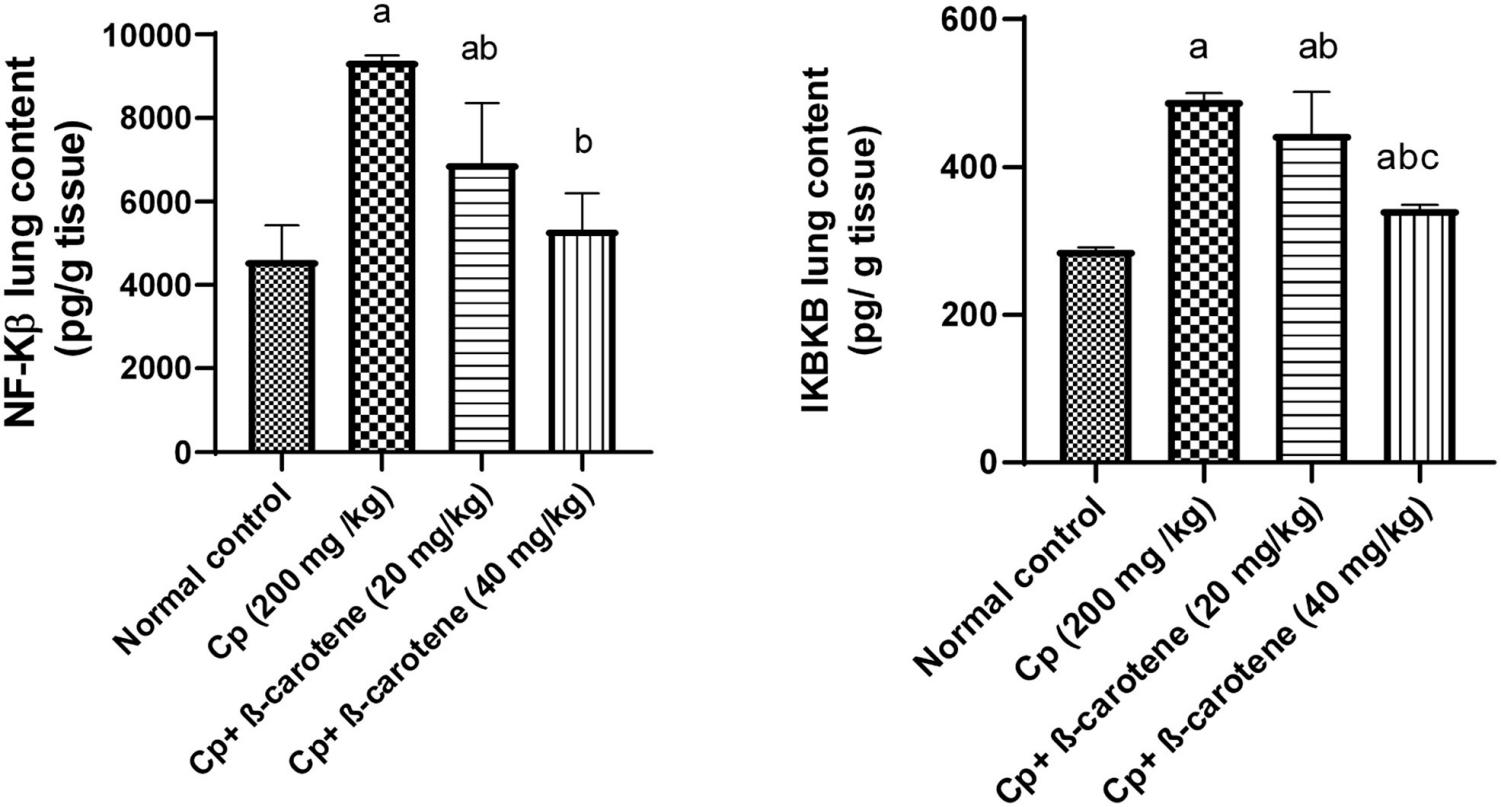
Effect of β-carotene on NF-κB and IKBKB lung contents. ^a^Significantly different from the control group. ^b^Significantly different from the CP group. ^c^Significantly different from the CP + β-carotene 20 mg/kg group at *P<0*.*05*.

Compared to the control group, administration of β-carotene (40 mg/ kg) in normal mice showed no change in the lung contents of NF-κB and IKBKB, while cyclophosphamide (CP) administration significantly (*P<0*.*05*) elevated NF-κB and IKBKB lung contents by 104% and 71%, respectively. However, administration of β-carotene 20 and 40 mg/ kg significantly (*P<0*.*05*) reduced NF-κB by 26% and 43%, and IKBKB by 9.2% and 71%, respectively, compared with that of the CP group (Fig 5).

### β-carotene supplementation lessened TNF-α, COX 2, and PKC lung contents

**Fig 6:**
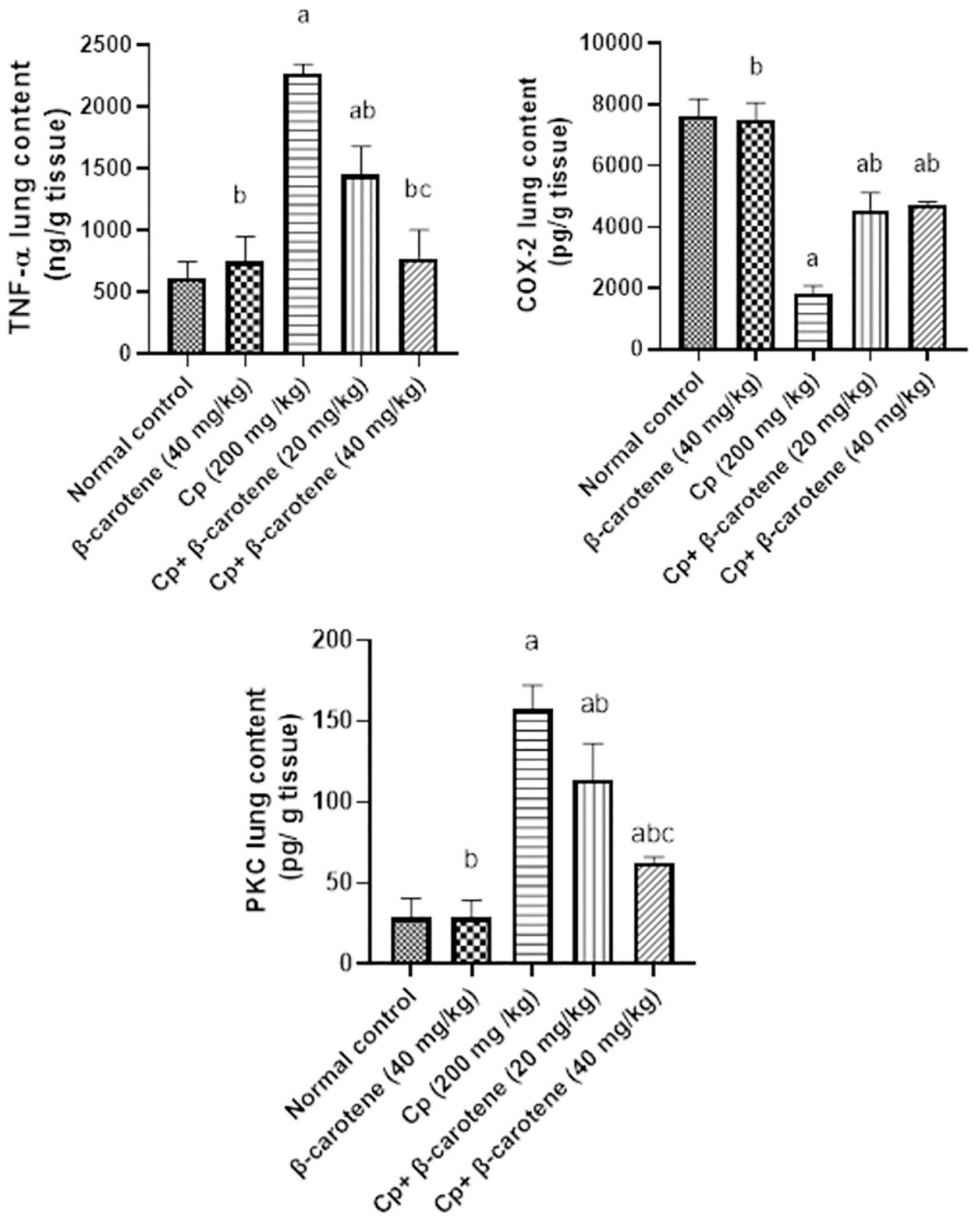
Effect of β-carotene on TNF-α, COX 2, and PKC lung contents. ^a^Significantly different from the control group. ^b^Significantly different from the CP group. ^c^Significantly different from the CP + β-carotene 20 mg/kg group at *P<0*.*05*.

Lung contents of TNF-α, COX-2, and PKC were not changed using β-carotene 40 mg/ kg in normal mice, however, they significantly (*P<0*.*05*) increased in CP- group by 270%, 320%, and 449%, respectively, comparing to the control group. While administration of β-carotene at 20 and 40 mg/ kg caused a significant (*P<0*.*05*) decline in the lung contents of TNF-α, COX-2, and PKC by 36%, 66%, 38%, and by 41%, 27%, and 60%, respectively, as compared to CP-group. Treatment with β-carotene at 40 mg/kg restores lung contents of TNF-α to their normal value, as compared to the CP group (Fig 6).

### β-carotene supplementation restores SIRT1 and PPARγ to their normal value

**Fig 7:**
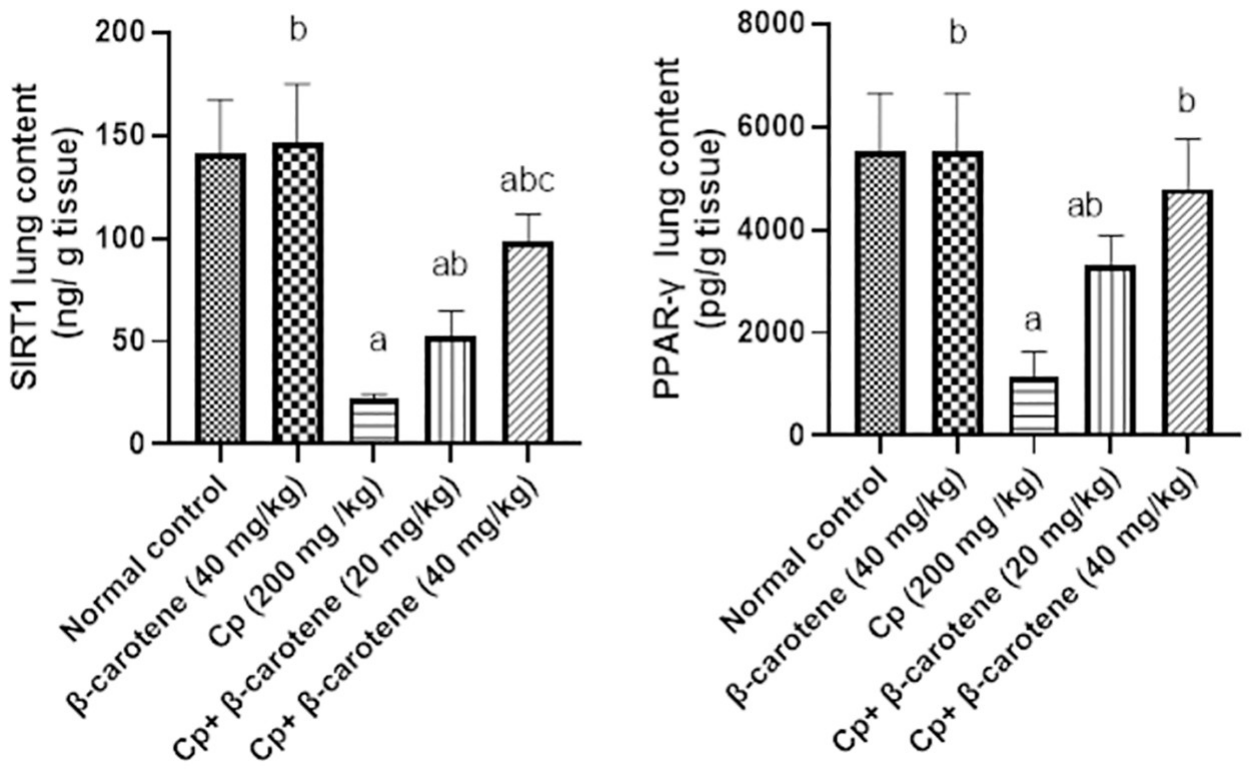
Effect of β-carotene on SIRT1 and PPARγ lung contents. ^a^Significantly different from the control group. ^b^Significantly different from the CP group. ^c^Significantly different from the CP + β-carotene 20 mg/kg group at *P<0*.*05*.

Compared to the control group, administration of β-carotene (40 mg/ kg) in normal mice showed no change in the lung contents of SIRT1 and PPARγ, while CP injection significantly (*P<0*.*05*) decreased the lung tissue contents of SIRT1 and PPARγ by 84% and 79%, respectively. Meanwhile, in β-carotene 20 and 40 mg/kg groups, SIRT1 and PPARγ contents significantly (*P<0*.*05*) increased by 139.4%, 350%, and 193%, 321%, respectively, as compared to CP-group. Treatment with β-carotene at 40 mg/kg returned lung contents of PPARγ to their normal value, as compared to the CP-group (Fig 7).

### β-carotene supplementation restores IL-17 to its normal value

**Fig 8:**
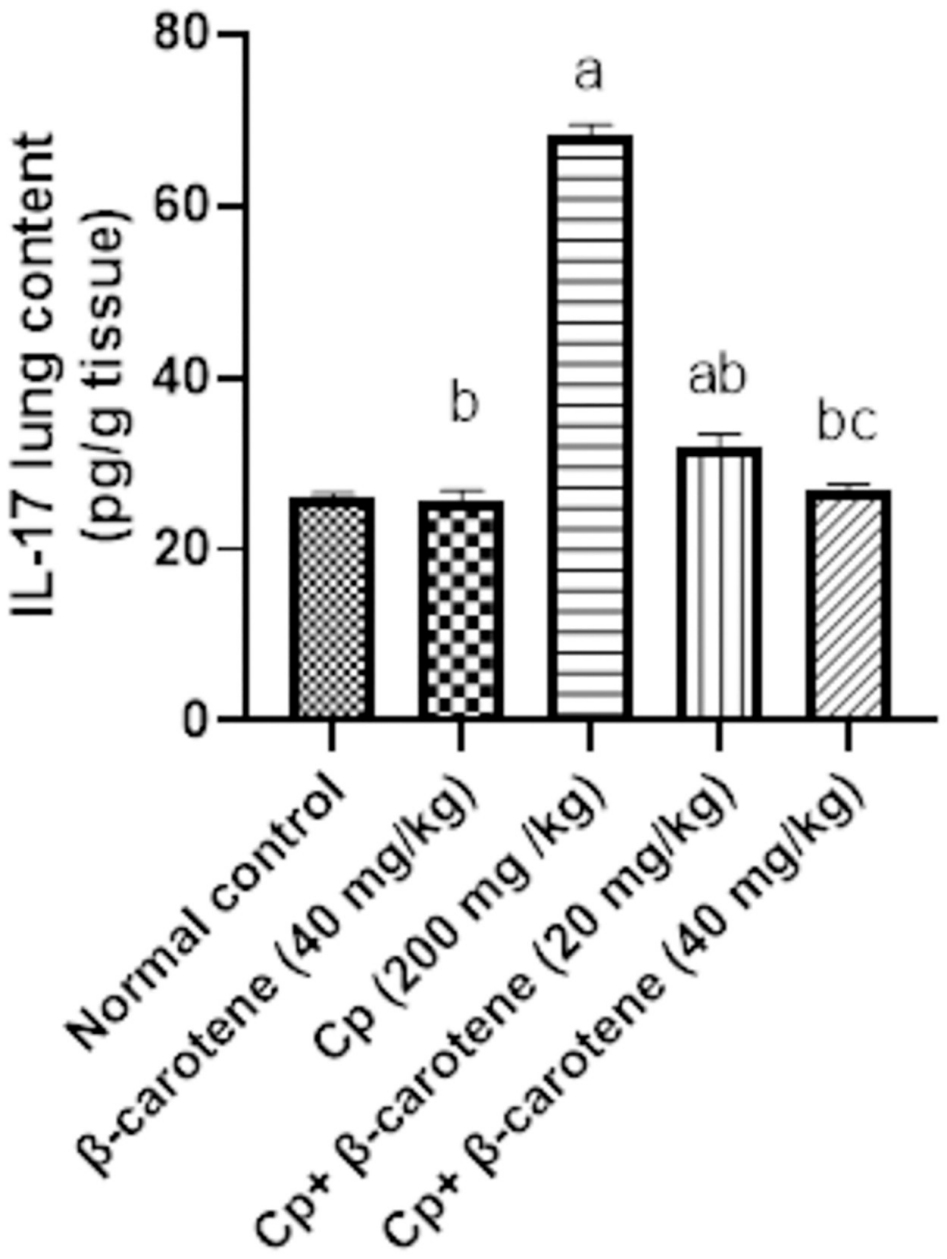
Effect of β-carotene on the IL-17 lung content. ^a^Significantly different from the control group. ^b^Significantly different from the CP group. ^c^Significantly different from the CP + β-carotene 20 mg/kg group at *P<0*.*05*.

Compared to the normal group, IL-17 lung content was not changed upon administration of β-carotene (40 mg/ kg) in normal mice, while CP administration significantly (*P<0*.*05*) elevated IL-17 lung content by 163%. Administration of β-carotene at 20 and 40 mg/kg significantly (*P<0*.*05*) reduced IL-17 by 53%, and 63%, respectively, compared with that of the CP group. Treatment with 40 mg/kg of β-carotene restores the lung content of IL-17 to its normal value, as compared to the CP-group (Fig 8).

### Histopathological findings

**Fig 9:**
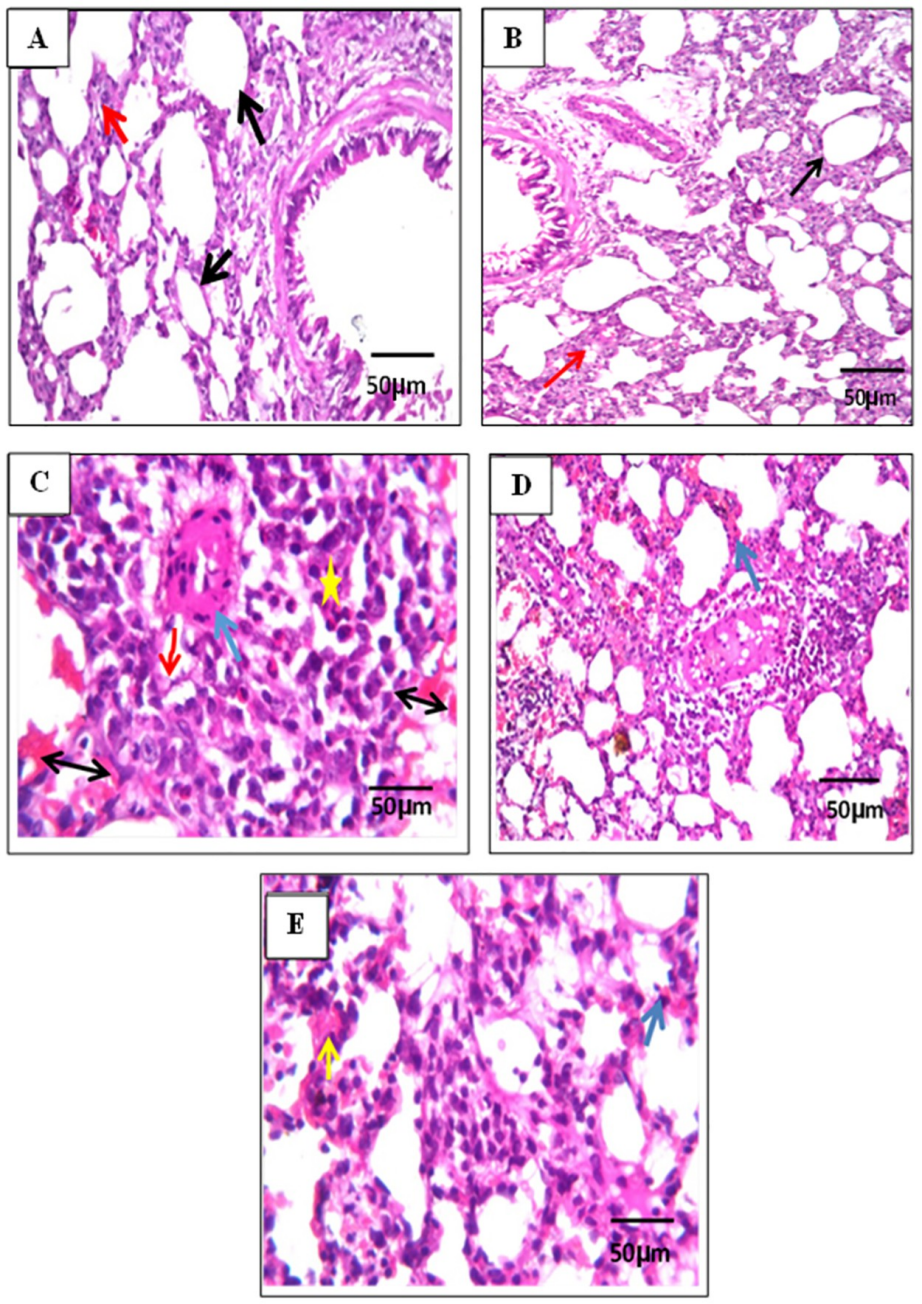
Effect of β-carotene on lung histopathology. Representative light microphotographs from the lung. There were no histopathological changes and the regular histological pattern of the blood vessels (black arrow) with normal alveolar septa (red arrow) and the surrounding air alveoli in the parenchyma were recorded in the control group (A). In the β-carotene 40 mg/ kg control group there were no histopathological changes and the regular histological pattern of the blood vessels (black arrow) with normal alveolar septa (green arrow) (B). However, in CP-group, there are thickened interalveolar septa (star), narrow alveoli (red arrow), and diffuse hemorrhages (blue arrow) were impacted the air associated with emphysema of some other air alveoli in some lobules of the parenchyma. There was multiple focal inflammatory cell aggregation in the parenchyma. The main inflammatory cells aggregation in the parenchyma were macrophages, lymphocytes, and eosinophils (arrow with two heads) (C). In β-carotene 20 mg/ kg groups, focal hemorrhages were detected in the air alveoli (blue arrow), associated with perivascular inflammatory cell infiltration (red arrow), and emphysema in the air alveoli (D). In β-carotene 40 mg/ kg groups, there was congestion in the blood vessels (blue arrow) of the parenchyma associated with mild inflammatory cells infiltration (yellow arrow) mainly lymphocytes and macrophages in between the ail alveoli (E) (H&E X 200).

Normal histological structure of the blood vessels with normal alveolar septa (red arrow) and air alveoli in the parenchyma was recorded in control and β-carotene 40 mg/kg control groups. However, in CP-group, there were thickened interalveolar septa, narrow alveoli, and diffuse hemorrhages that impacted the air associated with emphysema of some other air alveoli in some lobules of the parenchyma. There was multiple focal inflammatory cell aggregation in the parenchyma. The main inflammatory cells aggregation in the parenchyma were macrophages, lymphocytes, and eosinophils. In β-carotene 20 mg/ kg group, focal hemorrhages were detected in the air alveoli, associated with perivascular inflammatory cells infiltration, and emphysema in the air alveoli. In the β-carotene 40 mg/ kg group, there was only congestion in the blood vessels of the parenchyma associated with mild inflammatory cell infiltration mainly lymphocytes and macrophages in between the air alveoli (Fig 9).

### Histomorphometric results

**Fig 10:**
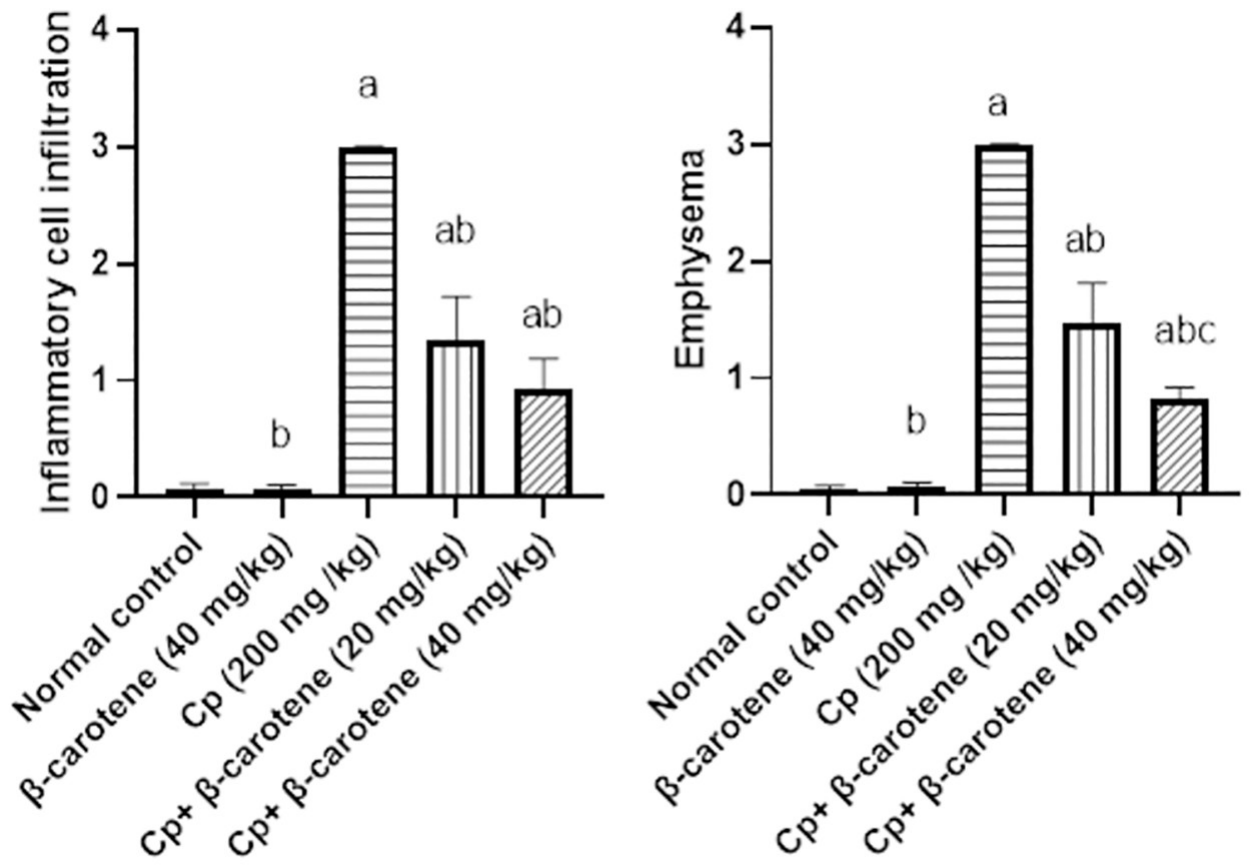
Effect of β-carotene on histomorphometric analysis. ^a^Significantly different from the control group. ^b^Significantly different from the CP group. ^c^Significantly different from the CP + β-carotene 20 mg/kg group at *P<0*.*05*.

Compared to the control and β-carotene control groups, the CP group recorded the highest score of inflammatory cell infiltration and emphysema. Alternatively, the administration of β-carotene 20 and 40mg/kg significantly (*P<0*.*05*) reduced the scoring number of inflammatory cell infiltration and emphysema by more than 70% when compared to the CP group (Fig 10).

### Discussion

The β-carotene was entirely purified from *S*. *obliquus* microalgae as an orange powder and identified by HPLC compared with its standard as well the chemical structure was confirmed by the ^1^H-NMR analysis. The ^1^H-NMR spectroscopic data and assignments of the isolated compound have corresponded to those stated for β-carotene [30, 31]. The ^1^H NMR data of the olefinic protons of β-carotene purified from *S*. *obliquus* microalgae showed the chemical shift of protons related to C-7 and 7′ at 6.18 ppm, C-8 and 8′ at 6.14 ppm, C-10, and 10′ at 6.27 ppm, C-11 and 11′ at 6.60 ppm, C-12 and 12′ at 6.37 ppm, C-14, and 14′ at 6.12 ppm, and C-15 and 15′ at 6.65 ppm. These chemical shifts agreed with the data of β-carotene isolated from the flowers of *Sesbania grandiflora* [31]. The spectrum signals of β-carotene are strongly overlapped due to the near co-occurrence of most of the chemical shifts [30].

Cyclophosphamide (CP), an immunosuppressive agent, provokes lung toxicity and other pathological patterns [12]. In this study, the CP group recorded the highest score of inflammatory cell infiltration and emphysema. This is related to the stimulation of macrophages, which accelerates the movement of the inflammatory cells from blood vessels into the alveolar space. Alveolar-capillary membranes are consequently injured, and the stimulated cells increase the permeability of lung vascular causing protein-rich lung edema [32]. On the other hand, β-carotene (20 and 40 mg/kg) administration attenuate the accumulation of CP inflammatory cell infiltration and emphysema. The high antioxidant ability of β-carotene inactivates discharge oxidants induced by CP and thus reduces the inflammatory cell infiltration and emphysema.

Upon CP administration, the CP-group in this study showed an induction of lung injury and disturbance in immunity via activation of macrophages and the release of high levels of proinflammatory cytokines NF-κB, IKBKB, TNF-α, COX-2, and PKC, low levels of SIRT1 and PPARγ, and high level of immunomodulatory cytokine IL-17, when compared with the control group. These results were in accord with that of El-Kholy et al. [33], who reported that CP administration produced pulmonary injury via an NF-kB/MAPK-dependent mechanism. CP also showed potent brain damage through inflammatory, oxidative, nuclear pyknosis, and neuron injury [34].

Administration of natural β-carotene isolated from *S*. *obliquus* in the present study showed a potent anti-inflammatory effect against CP-induced inflammation through the decrease of pro-inflammatory cytokines NF-κB, IKBKB, TNF-α, COX-2, and PKC. The potent antioxidant ability of β-carotene might be linked to these therapeutic properties. These findings are in accord with former studies that reported the protective effect of β-carotene against chronic bronchitis induced by long-term cigarette smoking through the reduction of different pro-inflammatory factors comprising IL-1α, IL-6, IL-8, and Gro I [35, 36]. In the animal model of carrageenan-induced inflammation, El-Baz et al. [4] also revealed the anti-inflammatory effect of β-carotene fraction of *Dunaliella salina* through the downregulation of proinflammatory cytokines IL-6, TNF-α, COX-2, and PGE2.

The natural β-carotene in the present study may exert an anti-inflammatory effect against CP- ALI in mice by inhibiting the NF-κB pathways, which are a primary regulator of proinflammatory cytokines. The present findings were supported by former results of Li et al. [37], who reported that β-carotene inhibited lipopolysaccharide (LPS)-induced inflammatory responses by suppressing TNF-α, IL-1β, IL-6, and NF-κβ expression. Besides, Bai et al. [38] stated that β-carotene suppresses NF-κB initiation and thereby decreases the expression of inflammatory genes in LPS-stimulated macrophages. Modulation of NF-κB modifies the inflammatory process provoked by cyclooxygenase-2 (COX-2) and pro-inflammatory cytokines [39].

The present result demonstrates the protective effect of β-carotene against CP- ALI in mice through the decline of PKC level, this agrees with Chichger et al. [40] who reported that inhibition of PKC is correlated with improvements in lung injury.

The natural β-carotene in the present study restored the lung content of IL-17 to its normal value when compared with the CP group, and this suggests the vital role of natural β-carotene to protect against autoimmune and inflammatory-related complications. The present result is in harmony with the earlier results of Trivedi and Jena [41], who showed that β-carotene administration decreased inflammation combined with ulcerative colitis in mice by lowering the colonic contents of immunomodulatory cytokines IL-17, IL-6, and TNFα. In the mouse model of vitamin A deficiency, supplementation of β-carotene also downregulated the levels of intestinal inflammatory cytokines, TNFα, IL1-β, and IL-6, and immunomodulatory cytokines, IL22, IL23, and IL17 [42]. Similarly, β-carotene controlled the contents of immunomodulatory cytokines IL-17 and IL-22, prospective regulators of prolonged inflammation in the intestinal tract [43].

The PPAR-γ hormone receptor plays an important role in modulating pulmonary inflammation and tissue injury or ischemia-reperfusion injury. It induced inhibition of NF-κB, cytokine, and eicosanoid production decreasing inflammatory cell influx and alveolar capillary injury [44].

The administration of natural β-carotene, in the present study, protected mice against CP-induced ALI and lung inflammation through the activation of the SIRT1 and PPARγ signaling pathway. SIRT1 expression leads to the down-regulation of antioxidant genes and stimulation of inflammation via modulating the FOXO1/NF-κβ signaling pathway [45]. The administration of lycopene, another carotenoid, protected against hepatic steatosis and inflammation in BCO2-expressing mice through the activation of SIRT1 and PPARγ signals [46]. The anti-inflammatory effect and immune responses to LPS stimulation of dietary lutein and polyunsaturated fatty acids were associated with modification of PPAR- α and γ, retinoic acid X receptor (RXR) α and γ and IL-1 mRNA amounts in the liver and the spleen of *Gallus gallus* chicken [47].

The natural β-carotene in the present study ameliorated histopathological pulmonary changes including the scoring number of inflammatory cell infiltration and emphysema produced by CP, suggesting the potent effect of β-carotene as an anti-inflammatory agent against CP-induced ALI in mice. In previous studies, β-carotene alleviated the histopathological injuries caused by chronic bronchitis induced by long-term cigarette smoking [35, 36].

## Conclusions

*Scenedesmus obliquus* microalgae is a good renewable source of β-carotene. The natural β-carotene isolated from *S*. *obliquus* at a 40 mg/kg dose showed a potent anti-inflammatory effect against CP-induced inflammation in mice through the decrease of NF-κB, IKBKB, IL-17, TNF-α, COX-2, and PKC. As well as it boosted the contents of SIRT1 and PPARγ in the lung tissues. β-carotene improved the histopathological changes induced by CP and reduced the scoring number of inflammatory cell infiltration and emphysema when compared to CP. The potent antioxidant ability of β-carotene is linked to these therapeutic properties. The present results suggest that natural β-carotene is a potential anti-inflammatory agent for different immune and inflammatory-related complications.
